# Types of Male Adolescent Violence Against Women in Three Contexts: Dating Violence Offline, Dating Violence Online, and Sexual Harassment Online Outside a Relationship

**DOI:** 10.3389/fpsyg.2022.850897

**Published:** 2022-03-09

**Authors:** María José Díaz-Aguado, Rosario Martínez-Arias

**Affiliations:** Unidad de Psicología Preventiva, Faculty of Psychology, Complutense University of Madrid, Madrid, Spain

**Keywords:** adolescence, violence against women, dating violence, online sexual harassment, justification of violence, risky online behaviors, self-esteem, risky online sexual behaviors

## Abstract

There has been little investigation of male adolescent violence against women as acknowledged by boys themselves, and even less on such violence in different contexts with comparative studies of behavior between those who perpetrate this violence and the population at large. This study used cluster analysis to establish a male adolescent typology based on boys’ self-reporting of violence against women in three contexts. The participants were 3,132 Spanish teenage boys aged 14–18 with experience of relationships with girls. Three discrete, identifiable types were obtained: the first group (69.8%), of *non-violent* boys; the second group (26%), more involved in *sexual harassment online* outside a relationship but with a low incidence of dating violence offline; the third group (4.2%), with *abuse in the three contexts* but less involved in sexual harassment online than the second group. The logistic regression analysis showed that justification of male dominance and violence was the main risk condition for inclusion in the second and third groups, followed by low self-esteem (for the third group) and risky sexual behaviors online (for the second and third groups). The findings based on these results are important for preventing male adolescent dating violence against women in the three male types detected.

## Introduction

As the United Nations (2021) recognizes, violence against women and girls is one of the most widespread, persistent and devastating human rights violations in our world today. It is a major obstacle to the achievement of the 2030 Agenda for Sustainable Development. It occurs worldwide, cutting across all generations, nationalities, communities, and spheres of our societies irrespective of age, ethnicity, disability, or other background.

### Male Dating Violence Against Women From a Gendered Perspective

Most of the violence against women is intimate partner violence. Worldwide, almost one third (27%) of women aged 15–49 who have been in a relationship report that they have been subjected to some form of physical and/or sexual violence by their intimate partner, and this figure is practically reached by the age of 15–19 (WHO, 2013). A study of 42,000 women in Europe that included the most frequent manifestations of this violence (such as psychological violence and control) estimated that around 43% of women aged 15–74 had suffered such violence in their lives (FRA, 2014).

Studies of adolescents show that male adolescent dating violence against women (DVAW) can appear in the earliest relationships they establish (Díaz-Aguado et al., 2021), can be a predictor of intimate partner violence in adulthood (Manchikanti Gómez, 2011), and relates to other forms of violence as bullying, with serious consequences for the subsequent development of both victims and perpetrators (Taquette and Monteiro, 2019).

Studies on male adolescent DVAW from a gendered perspective (Anderson, 2005; Santoro et al., 2018; Ferrer-Pérez and Bosch-Fiol, 2019) are scarce, and the few that exist are usually based on what the victims report to have suffered (Bradbury-Jones et al., 2019). Investigating DVAW based on self-reporting by adolescent boys is important, to know how different types of violence against women coalesce within adolescence, and their conditions of risk and protection, as well as helping to improve the efficacy of measures to prevent DVAW.

### Conditions of Risk and Protection in Violence Against Women From the Ecological Approach

As the majority of researchers on violence acknowledge, in order to eradicate violence and alleviate the damage it causes, it is important to consider the multiple contexts within which the risk and protection factors exist (Krug et al., 2002; Fulu and Miedema, 2015; Boonzaier and van Niekerk, 2021; Zinzow et al., 2021). The analysis of such violence carried out from this ecological perspective (Bronfenbrenner, 1979; Heise, 2011) considers characteristics from: (1) individuals; (2) interpersonal relationships or micro-system; (3) the community or meso-system; and (4) society or macro-system. As the ecological approach of human development recognizes, the influence of playing a social role (such as the role of traditional masculinity in a relationship with a women) is seen at different levels, including at the individual and micro-system levels with the interpersonal relationships that occur therein, but also at the community and the macro-system levels in which the role has its roots (Bronfenbrenner, 1979). This underlines the importance of representative research of the population to study violence against women in terms of the cultural context in which it occurs (Webster et al., 2019).

### Justification of Male Dominance and Violence Against Women

One of the main risk conditions for male DVAW from adolescence, in which the social macro-system and individual levels interact, is masculinity based on control and the use of violence to exercise it. In support of this argument is the fact that sexist attitudes and acceptance of violence are among the main predictors of the violence that some adolescent boys inflict on women (Shen et al., 2012; Díaz-Aguado and Martínez-Arias, 2015; Reyes et al., 2016; Bradbury-Jones et al., 2019; Taquette and Monteiro, 2019; Wesche and Dickson-Gomez, 2019), and that this violence diminishes with interventions designed to override the mentality the underlies it (Heise, 2011). The results from studies of adolescents in Spain also show that the mentality based on male dominance against women is the main risk condition for behaviors of maltreatment that boys report to have shown in their relationships with girls, evaluated from: identification with male dominance and violence against women, and the use of violence to resolve conflicts (Díaz-Aguado et al., 2011, 2014, 2020, 2021).

### Self-Esteem and Gender Role Stress

Studies on the emotional characteristics of male abusers show that men who exercise violence against women have lower self-esteem than men who do not (Gallagher and Parrott, 2011), which is supported by some studies on adolescents (Díaz-Aguado et al., 2011, Díaz-Aguado et al. 2013, 2021) but not by others (Foshee et al., 2001; Merino et al., 2021). This contradiction could be explained by the fact that the relation between self-esteem and aggression can vary according to age and type of study of adolescents, as well as the instrument applied to evaluate aggression (Teng et al., 2015).

One of the emotional problems that has received most attention in studies of male adolescent DVAW from the gendered perspective is the *gender role stress* caused by situations in which masculinity based on dominance is impeded from being exercised (Gallagher and Parrott, 2011; Baugher and Gazmararian, 2015; Reidy et al., 2015; Merino et al., 2021). It has even been confirmed that such masculine gender role stress (MGRS) predicts violence against women to a greater extent than other more cognitive components of sexism, both in adults (Jakupcak et al., 2002) and in adolescents aged 13–16 (Merino et al., 2021).

### Digital Technologies

The increasing use of digital technologies by adolescents has transformed all the environments in which we live and act, representing both an opportunity and a risk (Stonard, 2018), especially for those who tend to spend more time on their digital devices and act more impulsively when using them (Kim et al., 2020). A study across 11 countries in Europe by Durkee et al. (2016) found that 90% of adolescents with problematic internet use (PIU) presented multiple risk behaviors. As an example of the relation between PIU and male adolescent DVAW, it was found that boys who acknowledged perpetrating such violence against girls, and girls who reported having suffered this violence, both presented higher levels of PIU (Díaz-Aguado et al., 2021).

In addition, among the main risk conditions for male adolescent DVAW online include offline DVAW (Hinduja and Patchin, 2021) and stereotypical gender and dating beliefs (Reed et al., 2021).

### Sexual Harassment Online

One of the most widespread problems among adolescents who have grown up with digital devices is the reception of unwanted sexual solicitation, or sexual harassment online. One of the most significant studies on the prevalence of these behaviors in the European Union found that 11% of women older than 15 had been exposed to such problem; this was reported by double the figure of those aged 15–29 than in the 40–49 age group, and three times more than those aged 50–59 (FRA, 2014). The results of the study carried out in Spain on the generational differences in child sexual abuse remembered by adults aged 18–74 (Ferragut et al., 2021) reflect that while most forms of such abuse have decreased in the younger generation (18–24 years old), exposure to pornographic material and solicitations of personal photographs or videos of sexual content have increased, probably because they are exerted through the various digital devices that this generation uses much more frequently than the previous ones.

Based on these results, it is no surprise that studies on sexual harassment online in adolescents have multiplied in recent times, with findings that suggest it is one of most commonly perpetuated abuses, and that girls are the victims in the vast majority of cases (Baumgartner et al., 2010; Calvete et al., 2021; Díaz-Aguado et al., 2021; Mishna et al., 2021). This over-representation of girls among the victims of sexual harassment online leads us to consider it as a new form of violence against women (SHOAW), according to the definition of violence against women adopted by the Council of Europe (2011). In support of research on such violence from a gendered perspective, it should also be noted that the situations in which sexual harassment online occurs are perceived very differently by boys and girls; it has a far more negative emotional impact on girls, whereas some boys misconceive the severity of the impact of sexual harassment online on women (Brown et al., 2020).

Sexual harassment against women (SHAW) seems to be closely related to other forms of violence (as bullying) and risk behaviors that include browsing high risk websites and interacting with someone they have met online (Ybarra et al., 2007; Chang et al., 2016; Calvete et al., 2021).

### Typologies of Male Adolescents in Relation to Violence Against Women

Defining the typologies of adolescent boys in relation to the perpetration of different types of violence against women could be very useful for prevention. We found only two studies with this focus, both based exclusively on male adolescent DVAW. In the first, Lindhorst and Beadnell (2011) identified three patterns of exposure to violence in adolescent mothers: battered, moderate exposure, and low exposure. They concluded that typology is useful for illustrating an underlying “level of risk” in male adolescent DVAW, showing that psychological abuse might exist where physical abuse is absent, but that serious physical abuse exists in conjunction with psychological maltreatment. These findings were supported by research by Díaz-Aguado and Martínez-Arias (2015) whose probabilistic study of 4,147 boys in Spain aged 14–18 identified four discrete, identifiable groups. The first (76%) consisted of non-violent adolescent boys. The second (17%) comprised boys who isolated and controlled their partners. The third group (5%) was formed of boys who exerted only medium-level emotional abuse, and the fourth (2%) was represented by teenage boys who frequently engaged in all types of violence. In a logistic regression that compared the non-violent adolescents to the rest, the other groups presented lower self-esteem and greater justification of male dominance and intimate partner violence against women, greater justification of aggression in conflict resolution, as well as receiving more dominance and violence messages from adults in their family environment; they perceived violence against women as an abuse of lesser importance.

As these studies show, there has been little investigation of male adolescent violence against women as acknowledged by boys themselves, and less on such violence within different contexts, which include sexual harassment online outside the relationship with a partner, and comparative studies of behavior between those who perpetrate this violence and the population at large, as is the aim of this current research.

### The Male Adolescent Violence Against Women in Spain

To understand the context of this research, the rapid changes toward equality between men and women that have occurred in Spain in the last decades should be taken into consideration. These changes are especially noticeable in the awareness and rejection of violence that men inflict on women within an intimate relationship; to counter this phenomenon, a law was approved in 2004 (Ministry of Equality, 2004) that became an international reference in this field, and a cross-party parliamentary pact was established in 2017 to fight gendered violence. International recognition of the passing of this law came from bodies such as UN Women, the World Future Council and the Inter-Parliamentary Union, as one of the most effective regulatory frameworks worldwide for eradicating this type of violence.

The study on adolescent violence against women in Spain (Díaz-Aguado et al., 2021) showed that the most common situations girls reported experiencing were: emotional abuse (insulting or ridiculing, 17.3%), general abusive control (deciding for me, down to the smallest detail, 17.1%), and control by mobile phone (14.9%). The percentage of boys who acknowledge perpetrating all types of DVAW is much less than that of girls who report suffering them. For example, 2.3% of boys recognized hitting their partner, while 3.6% of girls acknowledged having received physical abuse; 3.1% of boys acknowledged pressuring girls to have sex against their will, against 11.1% of girls who reported this situation; 2.8% of boys reported sending messages *via* Internet or mobile phone in which they insulted, threatened, caused offense or attempted to scare their partner, whereas 6.3% of girls acknowledged receiving such messages.

The comparison of prevalence in the last decade (Díaz-Aguado et al., 2021) showed an evident increase in male adolescent DVAW in Spain between 2010 and 2013, which related to the emerging use of digital devices to perpetuate it, and the changes in the nature of relationships as a result of these technologies (Díaz-Aguado et al., 2014). In contrast, 2013–2020 saw a clear decrease in male adolescent DVAW in Spain, as in the previous period based on male violence reported by girls and on boys recognizing such actions. Such violence mainly took the form of psychological abuse and control, which diminished in the latter period thanks to awareness campaigns aimed at adolescents disseminated across the media from 2013 onward. These changes are closely linked to others detected in the same study between 2013 and 2020: among adolescents (less identification with the underlying mentality of gendered violence and greater identification with equality); in the family (fewer messages that support male domination of women, and more conversations between adults and adolescents on DVAW); at school (more activities to build equality and prevent gendered violence). The changes detected in this study (Díaz-Aguado et al., 2021) relate to other important advances toward equality between men and women, and the struggle against gendered violence presented between 2013 and 2020 in the macro-system in Spain, such as the greater scrutiny of this type of violence by the media, the 2017 cross-party parliamentary pact against gendered violence or the mass demonstrations in support of equality that took place on 8 March from 2018, which drew attention from the international press for their huge turnout.

The questionnaires used in 2020 (Díaz-Aguado et al., 2021) included for the first time questions on sexual harassment online perpetrated by a boy against a girl outside a relationship. The results showed that a high proportion of girls aged 14–20 reported being victims of this new type of abuse against women. The situations most frequently reported related to receiving (48%) or requesting sexually explicit photographs (43.9%) from boys; 23.4% of the girls surveyed said they had received requests for online cybersex. As in other forms of male adolescent DVAW, the percentage of boys who recognize having indulged in this type of behaviors is much less than that of girls who report suffering such aggression. The most frequently reported situation, recognized by 17.1% of boys, is requesting sexually explicit photographs online; 10.6% reported having sent girls sexually explicit photographs of themselves, and 7.4% had asked for online cybersex with a girl outside a relationship they were involved in.

The study found a significant relation between having experienced male adolescent DVAW and all the sexual harassment online situations outside the relationship that the participants were asked about in the survey (girls, whether they had experienced such situations, boys, if they had carried them out).

### Aims and Hypotheses

The main aims of this study were to define a typology of adolescent males in relation to violence against women that they reported having perpetrated in three contexts (dating violence offline, dating violence online, and sexual harassment online outside a relationship). Taking a general population sample enables us to understand how different contexts of violent behaviors combine, and their relation to conditions that can be modified through preventive interventions: justification of male dominance and violence against women, justification of violence for conflict resolution, stress resulting from difficulty to comply the traditional male role, problematic Internet use, risk behaviors online and self-esteem. As the survey was aimed at the general population, it was expected that there would be a substantial group of teenagers who showed no kind of violent behavior, and other groups that differed in the type and intensity of the violent behaviors they reported. The non-violent group that we expected to find would be used to compare to the groups that displayed violent behavior. Secondly, it was predicted that one or two groups would be identified as intermediate in terms of the types of violence perpetrated and their risk conditions. Finally, it was predicted that the survey would detect a small minority of adolescents that exercised violence against women in three contexts, with the highest scores for the various risk conditions and the lowest self-esteem.

## Materials and Methods

### Design and Participants

The study was designed around a probabilistic sample survey with stratified two-stage cluster sampling. The primary sampling unit was the school, and the secondary unit was the classroom. Depending on the school size, one, two or three full classrooms of students were randomly selected. The sample framework was the list of schools in Spain’s autonomous regions provided by the regions’ educational authorities. The sampling design was stratified by region and type of secondary education center (compulsory, academic, and vocational) with sizes proportional to the population sizes. In the Spanish education system, secondary education is divided into compulsory (12–16 years old) and non-compulsory (17–18 years old), the latter divided between academic and vocational. To determine the effective sample size controlling the possible effects of intra-school resemblance, an intra-class correlation of 0.10 was considered. In practice, the effect of school on the main variables (adolescent dating violence against women offline, adolescent dating violence against women online, and adolescent sexual harassment online) was less than 0.04 in all the relevant study variables (0.03 in harassment online, 0.02 in dating violence online, and 0.01 in dating violence offline). Consequently, the design effect was not corrected in the statistical analysis.

The initial sample consisted of 5,150 male adolescents, representative of all Spanish adolescents attending state or private schools of which those with no dating experience with girls (determined by an explicit question in the questionnaire) were not selected, as the objective was to study the dating relationships of boys with girls. The final sample included 3,132 Spanish teenage boys aged 14–18 (M age = 16.03, SD = 1.22). Concerning educational level, 57.4% were enrolled in compulsory secondary education, 29.5% in academic education, and 13.1% in vocational education. Regarding educational level of the parents, reported by the adolescents, 12.6% of the fathers and 9.6% of the mothers had completed an elementary level of education, 45.6% of the fathers and 44.6% of the mothers had completed secondary education, 25.8 of the fathers and 32% of the mothers had completed university education; 16% of the participants did not know the educational level of the father, and 13% of the mother. Considering the family situation, 77.9% lived with the father and mother.

The participants were enrolled at 251 secondary schools, 1,799 (57.1%) in compulsory education, and 1,353 (42.9%) in non-compulsory secondary education (918, or 29.1%, in academic, and 435, or 13.8%, in vocational). The mean number of participants per school was 20.2, ranging from 8 to 90, with a median of 18. The number of participants attending state schools was 1,984 (63.5%), and the participants from private schools numbered 1,138 (36.5%). A total of 2,875 students (91.8%) reported that they were native-born.

### Procedure

The principals of the schools selected were informed of the study and their participation requested.

Passive consent procedures were employed in accordance with recommended ethical guidelines. Parents of students under 18 had the opportunity to refuse consent for their child’s participation by returning a written form to the school office. Informed consent was requested from the 18-year-old students. All students were instructed that the survey was voluntary, they could withdraw at any time, and that their responses were anonymous.

Data collection at the school was carried out *via* Internet. A teacher remained in the room as the survey was administered to answer questions and resolve potential computer problems. The average time required to complete the questionnaire was about 50 min.

### Measures

All measures were used and validated in previous research (Díaz-Aguado et al., 2011, 2021), and the technical aspects and detailed psychometric properties of the measures are available for inspection.

#### Male Adolescent Dating Violence Against Women Offline

A questionnaire was drawn up composed of 11 indicators that referred to different forms of aggression toward women: physical, relational and emotional. The indicators were: (a) insults; (b) humiliation; (c) trying to isolate the girl from her friends; (d) trying to control her behavior and decisions; (e) scaring her; (f) physical aggression; (g) threats of aggression to force her to do things; (h) verbal intimidation, insults or behaviors of a sexual nature; (i) pressure to perform sexual; acts (j) accusing her of provoking the violence inflicted on her in any of the above situations; (k) trying to control her *via* mobile phone. The response format was a four-point Likert-type scale: never, sometimes, frequently, many times. An exploratory factor analysis based on polychoric correlations was carried out with FACTOR 10.10 software (Lorenzo-Seva and Ferrando, 2020). An unweighted least squares extraction produced one identifiable factor explaining 83% of the variance, and with loadings greater than 0.70. The Cronbach alpha coefficient for the 11 items was 0.92. The summative score of the 11 items was used.

#### Male Adolescent Dating Violence Against Women Online

This was evaluated by six indicators with a four-point Likert-type response format: never, sometimes, frequently, many times. The items referred to types of messages sent to the partner *via* Internet: (a) ridiculing her; (b) insults; (c) scaring her; (d) threats of aggression to force her to do things; (e) dissemination of images of her of a sexual nature without permission; (f) pressure on her to perform sexual acts. Exploratory factor analysis based on polychoric correlations carried out with FACTOR 10.10 software with unweighted least squares extraction produced one identifiable factor explaining 93% of the variance, and with loadings greater than 0.80. The alpha coefficient for these factor was 0.98. The summative score of the six items was used.

#### Male Adolescent Sexual Harassment Against Women Online

The Tynes et al. (2010) online victimization scale for adolescents was adapted to gauge online sexual harassment against women outside a relationship; it contained six items, with a four-point Likert-type response format: never, sometimes, frequently, many times. The items were: (a) I have asked her to cybersex online; (b) I have continued to communicate with her even after she told me to stop; (c) I have spread rumors about her sexual behavior online; (d) I have asked her for sexy photographs of herself online; (e) I have shown her sexual images online; (f) I have sent her unsolicited sexual content in emails or messages. Exploratory factor analysis based on polychoric correlations carried out with FACTOR 10.10 software with unweighted least squares extraction produced one identifiable factor explaining 73% of the variance, and with loadings greater than 0.70. The alpha coefficient of the data from the sample was 0.76.

#### Self-Esteem

We used the 10 items in the self-esteem scale (Rosenberg, 1965) widely used in the study of this construct, and whose psychometric features have been studied in depth worldwide. Validation studies support the one-dimensional nature of this scale, which exhibits an internal consistency of 0.82 in the study sample. The extent of agreement with the statements was scored on a four-point Likert scale, 1 for totally disagree, 4 for totally agree.

#### Justification of Male Dominance and Violence

A scale to measure justification of male dominance, male IPV against women in a relationship, and justification of violence as a way to resolve conflicts, consisting of 10 Likert-type items with 4 points (1–4) was used (Díaz-Aguado and Martínez-Arias, 2015). An unweighted least squares extraction and Promin rotation showed two factors. The first factor of seven items could be interpreted as “justification of male dominance and violence against women” (JMDVAW) (α = 0.76): “For the sake of her children, a women who puts up with violence from her husband or partner should not report him to the police”; “If a woman has been abused by her partner, she must have done something to provoke him”; “A proper father should make his family know that he is the boss”; “If a woman is battered by her partner and she does not leave him, it must surely be because she is not entirely unhappy in such a situation”; “For a relationship between a man and a woman to prosper, the woman should avoid contradicting her partner”; “The violence that takes place at home is a family matter and should be kept in the family”; “A man is justified in assaulting his wife or girlfriend when she decides to leave him.” The second factor consisted of three items referred to as “justification of violence as conflict resolution” (JVCR) (α = 0.77): “An assault on someone is justified if they have taken something that was yours”; “It is right to threaten someone in order to let them know who the boss is”; “It is right to hit someone who has offended you.”

#### Masculine Gender Role Stress

This was evaluated by two of the five factors on the Eisler and Skidmore (1987). Factors 3 and 4 were selected as being the most relevant to the teenagers in our study. Factor 3, *subordination to women (SW)*: situations that place one in the position of being outperformed by women. This consists of 8 items, such as: “being with women who are more successful or who make more money than you.” Factor 4, *intellectual inferiority (II):* situations that question one’s rational abilities or demonstrate one’s uncertainty, lack of ambition or indecisiveness. This consists of 7 items, such as: “having to ask for directions when you are lost.” The responses to each item were scored on a Likert scale of 0–5, according to which the participant believes he would feel no anxiety or extreme anxiety in the situation. The alpha coefficient of the data from the sample was 0.85 for the scale referring to subordination to women, and 0.78 for the intellectual inferiority scale. These scales were validated and adapted to the Spanish population by Merino (2018). The responses to each item were scored on a Likert scale of 0 to 5, according to which the participant believes he would feel no anxiety or extreme anxiety in the situation. The alpha coefficient of the data from the sample was 0.86 for the scale referring to subordination to women, and 0.81 for the intellectual inferiority scale.

#### Problematic Internet Use

The Caplan (2010) “generalized problematic Internet use scale 2” (GPIUS2) was used to assess problematic Internet use. The scale was translated into Spanish and validated in previous research (Díaz-Aguado et al., 2021). The scale can be used in two different ways, as a set of separate sub-scales or as a summative total score of the 15 items. This summative score was used. The alpha coefficient of the data from the study sample was 0.88.

#### Risky Online Behavior

This scale was initially constructed according to indicators proposed by Ybarra et al. (2007) on four types of risky online behavior: (1) disclosure of personal information, (2) aggressive behavior; (3) interacting with someone you have met online and, (4) risky sexual online behavior. Díaz-Aguado et al. (2021) validated the scale used here for Spain. Exploratory factor analysis was carried out with FACTOR 10.10 software (Lorenzo-Seva and Ferrando, 2020), using unweighted least squares and Promin rotation as extraction method. Three correlated factors were obtained (with a range of values between 0.59 and 0.71) that explained 56% of the variance. Factor 1, *disclosure of personal information* (DPI), with 6 items: giving your first name and surnames to a stranger, giving your home address, accepting a stranger as an online friend, giving the name of your school, giving your age and location. Factor 2, *risky sexual online behavior (RSOB)*, with six items, three risky behaviors involving sexting and three behaviors relating to interacting with someone you have met online: “Post or send a highly sexual photograph of me”; “post or send a sexual photograph of my partner”; “post or send a photograph of me that my parents would not approve of”; “talk about sex with someone I have met on Internet”; “arrange to meet someone I have met on Internet”; “use webcam or mobile phone camera when I communicate with a stranger.” The alpha coefficient for the set of six items was 0.72. Factor 3, *aggressive behavior and visiting risky websites (ABVRW)*, with six items: “Send messages that insult or offend people”; “Call someone to upset them”; “Respond to a message that has insulted or offended me”; “Browse a website that my parents would not approve of”; “Browse websites that show violence”; “Browse websites that show sexual acts.” The alpha coefficient for this set of six items was 0.74.

### Data Analysis

Data preparation was carried out using IBM SPSS v.27 software. All analyses were made with SPSS v.27, except for the exploratory factor analysis of some scales that were done with FACTOR v.10.10 software (Lorenzo-Seva and Ferrando, 2020).

Summary scores for some factors were obtained by adding up the scores for the items of the corresponding factors and dividing them by the number of items to maintain the scores within the original scale (self-esteem: 1–4, MGRS-II and MGRS-SW: 0–4, JMDVAW and JVCR: 1–4).

Before computing the summary scores, the items’ missing values were imputed using IBM SPSS v.27 software with the Expectation-Maximization (E-M) algorithm. The procedure is iterative and other variables are used to attribute a value (Expectation) followed by checks to see whether that is the most likely value (Maximization). If not, it re-imputes a more likely value. This continues until the most likely value is reached (Enders, 2010).

The main procedure used for the data analysis was two-step clustering that classifies individuals into groups based on their patterns of responses to sets of observed variables. It is an exploratory multivariate data analysis that identifies similar groups of objects within data sets with both continuous and categorical variables (Norusis, 2009). This study used the likelihood distance measure as the similarity criterion, and the Schwarz Bayesian Information Criterion (BIC) to determine the optimal number of clusters. Two-step cluster analysis also calculates the silhouette coefficient, which measures the quality of the clustering solution. Validity is established based on a combination of two different measures: cohesion, which is the closeness between the members of the same cluster, and separation, the closeness between the members and the centroids of the different clusters. The procedure is performed in two distinct phases. In the first step, a sequential clustering procedure is used to create pre-clusters by scanning the data case by case and assigning them to a previously formed cluster or a new one, based on the distance criterion. When all cases have been assigned to a pre-cluster, all objects in the same pre-cluster are treated as a single entity to reduce the size of the matrix that contains distances between all possible pairs of cases (Norusis, 2009). The second step consists of applying a hierarchical clustering algorithm to the pre-clusters and defining the best number of clusters based on a BIC criterion. Clusters were formed from three standardized quantitative variables: DVAW offline, DVAW online, and SHAW online.

Due to the lack of compliance with the assumptions of the parametric tests, the relationship between cluster membership and quantitative variables was examined by the Kruskal–Wallis non-parametric test with all pairwise comparisons; significance values were adjusted by the Bonferroni correction for multiple tests. Statistical significance for the comparisons was set at 0.01 due to the large sample size.

Rosenthal’s *r* statistic, with values between 0 and 1 (Rosenthal, 1991), was used to compute the effect sizes of the pairwise comparisons.

Finally, the variables that showed statistically significant differences among groups were entered as predictors in a multinomial logistic regression analysis.

## Results

Table 1 shows the correlations between variables used in the formation of clusters (triangular lower matrix) and the descriptive statistics. All relationships for variables included in the cluster analysis were positive and significant (*p* < 0.001).

**TABLE 1:** Correlations between variables used for the formation of clusters and descriptive statistics.

	Mean (SD)	Median	DVAW offline	DVAW online	SHAW online
DVAW offline	0.65 (2.77)	0.00	–		
DVAW online	0.15 (0.86)	0.00	0.21***	–	
SHAW online	1.13 (2.43)	0.00	0.28***	0.08***	–

*DVAW, dating violence against women; SHAW, sexual harassment against women.*

***p < 0.001.

The variables were previously standardized. The solution selected following the Schwarz BIC criterion was the three-cluster solution, which provided a good solution (close to 0.80, with 1.00 being the maximum value) in cohesion and separation. All variables were important in the cluster formation.

The number of subjects in the clusters was 2,200 (69.8%), 821 (26.0%), and 131 (4.2%) for clusters 1, 2, and 3, respectively. Table 2 shows the main results for the three variables in the clusters (Mean, Standard Deviation, Range, and Median), and the statistical significance of the differences.

**TABLE 2:** Descriptive statistics of cluster variables and significant of the differences among clusters.

	Cluster 1: non-violent		Cluster 2: sexual harassment online		Cluster 3: abuse in the three contexts		Pairwise, ES (r)
				
	Mean (SD) (Range)	Median	Mean (SD) (Range)	Median	Mean (SD) (Range)	Median	
DVAW offline	0.20 (0.72) (0–9)	0.00	0.54 (1.18) (0–9)	0.00	8.85 (9.86) (0–33)	5.00	(C1 < C2)***, ES = 0.18
							(C1 < C3)***, ES = 0.47
							(C2 < C3)***, ES = 0.59
DVAW online	0.01 (0.02) (0–1)	0.00	0.01_a_ (0.03) (0–1)	0.00	3.58 (2,38) (0–6)	5.00	(C1 = C2) ns
							(C1 < C3)***, ES = 0.58
							(C2 < C3)***, ES = 0.85
SHAW online	0.06 (0.38) (0–5)	0.00	3.72_b_ (3.02) (1–18)	3.00	2.93 (4.29) (0–18)	1.00	(C1 < C2)***, ES = 0.52
							(C1 < C3)***, ES = 0.58
							(C2 > C3)***, ES = 0.31

*DV, dating violence against women; SHAW, sexual harassment against women.*

ES (r), Rosenthal’s r Effect Size; ***p < 0.001.

The Kruskal–Wallis (K–W) test with two degrees of freedom showed statistically significant differences for each of the three variables used in cluster formation. Results (K–W) and for each of the comparisons with the Bonferroni correction: DVAW online (K–W = 2,407.53, *p* < 0.001); DVAW offline (K–W = 596.76, *p* < 0.001); SHAW online (K–W = 2,614.79, *p* < 0.001). Pairwise statistically significant differences adjusted by the Bonferroni correction are presented in Table 2, as well as the effect sizes.

According to the results in Table 2, members of the first group had means close to 0 and medians of 0 on all variables, and based on the response pattern, this group was termed *non-violent* because almost none of the members had inflicted abusive behavior on their female partners or online sexual harassment of girls outside a relationship. There are two groups of adolescents who reported having exercised abusive behaviors to some extent. Members of the second group had exercised DVAW offline sometimes, but to a much lesser extent than group 3. This group stands out against the other two in SHAW online. We named this group *sexual harassment online.* Group 3 of male adolescents scored high in violence against women in all three of the contexts, and based on this response pattern it was defined as *abuse in the three contexts.*

Possible differences between clusters in terms of age and type of education were explored. Age revealed significant differences among groups with a very small effect size, *F*(2,3149) = 9.98, *p* < 0.001, η*^2^* = 0.006. The Bonferroni *post hoc* contrast showed that subjects in group 2 were older than those in groups 1 and 3 (Ms = 15.98, 16.19, 15.95; *p* < 0.05). The chi-squared contrast showed no significant association between class and type of education, χ^2^ (4, 3152) = 9.68, *p* = 0.06, Cramer’s V = 0.03.

The covariates with the cluster membership were those mentioned in the hypotheses: self-esteem, justification of dominance and violence (JMDVAW and JVCR), masculine gender role stress (as WS and II), PIU and the three factors of online risk behaviors (DPI, RSOB, and ABRW).

Table 3 shows the descriptive statistics for covariates, correlations, and collinearity. The collinearity statistics are within the established limits.

**TABLE 3:** Descriptive statistics of covariates, correlations, and collinearity statistics.

	Mean (SD) and (Range)	Self-es	MGRS-II	MGRS-WS	JMDVAW	JVCR	PIU	DPI	RSOB	ABVRW	Collinearity statistics	
											Tolerance	VIF
Self-Es	3.16 (0.60) (1–4)	–									0.88	1.14
MGRS-II	0.62 (0.68) (0–4)	−0.214**	–								0.65	1.53
MGRS-WS	0.37 (0.59) (0–4)	−0.079**	0.605**	–							0.59	1.70
JMDVW	1.19 (0.40) (1–4)	−0.097**	0.332**	0.510**	–						0.72	1.40
JVCR	1.44 (0.50) (1–4)	–0.044	0.303**	0.495**	0.587**	–					0.70	1.43
PIU	15.83 (10.9) (0–60)	−0.294**	0.303**	0.202**	0.153**	0.172**	–				0.75	1.33
DPI	8.96 (5.22) (0–18)	0.024	0.077**	0.059**	0.024	0.087**	0.216**	–			0.76	1.32
RSOB	2.78 (3.30) (0–18)	−0.086**	0.135**	0.160**	0.163**	0.227**	0.284**	0.392**	–		0.77	1.30
ABVRW	10.16 (5,51) (0–18)	–0.031	0.156**	0.146**	0.083**	0.253**	0.360**	0.460**	0.475**	–	0.71	1.41

*Self-es, Self-esteem; MGRS-II, Masculine gender role stress- intellectual inferiority; MGRS-WS, Masculine gender role stress-women subordination; JMDVAW, Justification of male dominance and violence against women; JVCR, Justification of violence as conflict resolution; PIU, Problematic internet use; DPI, disclosure personal information; RSOB, risky sexual online behavior; ABVRW, aggressive behavior and visiting risky website; VIF, Variance Inflation Factor.*

**p < 0.01.

Table 4 presents the main results for the covariate variables in the clusters (Mean, Standard Deviation, and Median), the statistical significance of the pairwise comparisons and effect sizes.

**TABLE 4:** Descriptive statistics of covariates in the three clusters, pairwise comparisons and Effect Sizes.

Covariates	Cluster 1: non-violent		Cluster 2: sexual harassment online		Cluster 3: abuse in three contexts		Pairwise comparisons, Median Test, ES (r)
	Mean (SD)	Median	Mean (SD)	Median	Mean (SD)	Median	
Self-es	3.19 (0.59)	3.30	3.12 (0.61)	3.20	2.82 (0.62)	2.80	(C1 = C2)^ns^, ES = 0.05 (C1 > C3)***, ES = 0.14
							(C2 > C3)***, ES = 0.17
MGRS-II	0.54 (0.60)	0.33	0.74 (0.74)	0.61	1.24 (1.06)	1.00	(C1 < C2)***, ES = 0.12 (C1 < C3)***, ES = 0.17
							(C2 < C3)***, ES = 0.16
MGRS-WS	0.31 (0.48)	0.11	0.46 (0.64)	0.22	1.02 (1.11)	0.67	(C1 < C2)***, ES = 0.14 (C1 < C3)***, ES = 0.20
							(C2 < C3)***, ES = 0.16
JMDVAW	1.14 (0.29)	1.00	1.23 (0.46)	1.10	1.74 (0.83)	1.43	(C1 < C2)***, ES = 0.10 (C1 < C3)***, ES = 0.23
							(C2 < C3)***, ES = 0.19
JVCR	1.36 (0.42)	1.17	1.57 (0.54)	1.50	1.96 (0.81)	1.83	(C1 < C2)***, ES = 0.19 (C1 < C3)***, ES = 0.20
							(C2 < C3)***, ES = 0.12
PIU	14.27 (10.23)	13.00	19.02 (11.17)	18.00	21.88 (12.93)	21.00	(C1 < C2)***, ES = 0.19, (C1 < C3)***, ES = 0.14
							(C2 = C3)^ns^, ES = 0.07
DPI	8.25 (5.13)	8.00	11.50 (4.73)	12.00	10.11 (4.90)	11.00	(C1 < C2)***, ES = 0.26 (C1 < C3)**, ES = 0.08
							(C2 = C3)^ns^, ES = 0.04
RSOB	1.71 (2.35)	1.00	5.50 (4.06)	5.00	4.73 (4.41)	4.00	(C1 < C2)***, ES = 0.43 (C1 < C3)***, ES = 0.18
							(C2 = C3)^ns^, ES = 0.01
ABVRW	9.14 (5.32)	9.00	13.41 (4.71)	14.00	12.25 (5.59)	13.00	(C1 < C2)***, ES = 0.32 (C1 < C3)***, ES = 0.13
							(C2 = C3)^ns^, ES = 0.01

Self-es, Self-esteem; MGRS-II, Masculine gender role stress- intellectual inferiority; MGRS-WS, Masculine gender role stress-women subordination; JMDVAW, Justification of male dominance and violence against women; JVCR, Justification of violence as conflict resolution; PIU, Problematic internet use; DPI, disclosure personal information**;** RSOB, risky sexual online behavior; ABVRW, aggressive behavior and visiting risky website. ***p < 0.001; **p < 0.01; ns, non-significant.

The K–W test with two degrees of freedom showed statistically significant differences between clusters in all covariates: Self-Esteem (K–W = 51.17, *p* < 0.001), MGRS-II (K–W = 100.58, *p* < 0.001), MGRS-SW (K–W = 134.56, *p* < 0.001), JMDVAW (K–W = 149.44, *p* < 0.001), JVCR (K–W = 184.04, *p* < 0.001), PIU (K–W = 149.89, *p* < 0.001), DPI (K–W = 213.46, *p* < 0.001), RSOB (K–W = 605.96, *p* < 0.001), ABVRW (K–W = 330.48, *p* < 0.001).

The comparisons of medians tests were also carried out, with results similar to those of the K–W tests, so these are not presented here.

The differences were statistically significant between the three groups in MGRS-II, MGRS-SW, JMDVAW, and JVCR, all showing the same pattern: cluster 1 < cluster 2 < cluster 3. No significant differences in self-esteem were found between groups 1 and 2, both surpassing group 3. In variables related to Internet use, no differences were found between cluster 2 and cluster 3. Both clusters had higher scores for PIU and risky online behaviors than cluster 1.

The results from the multinomial logistic regression are presented in Table 5. The reference for comparison was the *non-violent group* (cluster 1). The alpha for significance was set at.01. Variables that were statistically significant for the prediction (*p* < 0.01) were self-esteem, justification of violence against women (JMDVAW) and the three factors of risky online behaviors (DPI, RSOB, and ABVRW). The final model was statistically significant, χ2 (18) = 915.87, *p* < 0.001, indicating that at least one of the predictors in the model was not equal to zero. The Nagelkerke pseudo *R*^2^ value was 0.35.

**TABLE 5:** Multinomial logistic regression with cluster membership as dependent variable.

	B (SE)	95% IC for odds ratio		
		Lower	Odds ratio	Upper
**Cluster 2. Sexual harassment online**				
Intercept	−4.03 (0.39)***			
Self-es	0.01 (0.09)	0.83	0.99	1.18
MGRS-II	0.67 (0.95)	0.89	1.07	1.29
MGRS-WS	0.01 (0.12)	0.79	1.00	1.26
JMDVAW	0.36 (0.17)*	1.02	1.43	1.99
JVCR	0.13 (0.13)	0.89	1.14	1.48
PIU	0.01 (0.005)	1.00	1.01	1.02
DPI	0.04 (0.012)***	1.02	1.04	1.07
RSOB	0.29 (0.02)***	1.28	1.33	1.38
ABVRW	0.06 (0.012)***	1.03	1.06	1.08
**Cluster 3: Abuse in three contexts**				
Intercept	−4.69 (0.65)***			
Self-es	−0.58 (0.16)***	0.42	0.57	0.77
MGRS-II	0.28 (0.16)	0.95	1.28	1.75
MGRS-WS	0.25 (0.17)	0.91	1.25	1.76
JMDVAW	1.08 (0.22)***	1.91	2.77	4.26
JVCR	0.36 (0.22)	0.94	1.44	2.20
PIU	0.02 (0.01)	1.00	1.02	1.04
DPI	0.02 (0.02)	0.97	1.01	1.07
RSOB	0.20 (0.03)***	1.15	1.23	1.31
ABVRW	0.02 (0.02)	0.98	1.03	1.07

*Cluster 1 is the reference class.*

*P < 0.05, **p < 0.01, ***p < 0.001.

*Self-es, Self-esteem; MGRS-II, Masculine gender role stress-intellectual inferiority; MGRS-WS, Masculine gender role stress-women subordination; JMDVAW, Justification of male dominance and violence against women; JVCR, Justification of violence as conflict resolution; PIU, Problematic internet use; DPI, disclosure personal information**;** RSOB, risky sexual online behavior; ABVRW, aggressive behavior and visiting risky website.*

The odds ratios indicated the predicted change in the odds of membership of a particular class compared with the *non-violent* group (cluster 1) for a one-standard deviation increase in the covariate, with all other variables in the model remaining constant.

First, predictors of membership of the *sexual harassment online* cluster, compared with the *non-violent* cluster, were considered. An increase of one standard deviation in JMDVAW increased the odds of membership of the *sexual harassment online* cluster by 43%, in the case of DPI by 4%, in RSOB by 33%, and in the case of ABVRW by 6%.

Next, predictors of membership of cluster 3, *abuse in the three contexts*, compared with the *non-violent* cluster, were considered. Increases of one standard deviation in self-esteem decreased the odds of membership in cluster 3 by 57%. By contrast, an increase of one standard deviation in JMDVAW increased the odds of membership by 177%, and RSOB by 23%.

## Discussion

Three discrete, identifiable types were obtained from the two-step cluster analysis based on violence against women that the boys reported having perpetrated in three contexts: dating violence offline, dating violence online, and sexual harassment online outside a relationship. As expected, the biggest group (69.8%) consisted of *non-violent* adolescent boys; the third group, a small minority of 4.2% of those surveyed, with *abuse in the three contexts*, and was formed of male adolescents who frequently exercised violence against women within a relationship in both offline and online scenarios, as well as online sexual harassment. The analysis also confirmed the existence of an intermediate group (26%), with a low incidence of DVAW offline and no DVAW online. It was not expected that this intermediate group would present with more *sexual harassment online* outside a relationship than the group that corresponded to abuse in the three contexts. The identification of this group with *sexual harassment online* and its risk conditions is one of the main contributions of this research.

The differences between the three groups in terms of the risk conditions evaluated provide important information on how to proceed to bolster prevention. According to predictions, in the four variables related to the underlying mentality behind violence against women (JMDVAW, JVCR, MGRS-WS, and MGRS-II), the group of abuse in the three contexts registered significantly higher scores than the group associated to *sexual harassment online*, which in turn scored higher than the *non-violent* group. However, the regression analysis results emphasize that the first of these indicators, the mentality that justifies male dominance and violence against women, is the strongest predictor of the violence perpetrated against them, especially for membership of the group that reports inflicting more violence on their partner and also, though to a lesser extent, of the group associated with more sexual harassment online outside the relationship. These results are in line with those in previous research on DVAW (Shen et al., 2012; Díaz-Aguado and Martínez-Arias, 2015; Reyes et al., 2016; Bradbury-Jones et al., 2019; Taquette and Monteiro, 2019; Wesche and Dickson-Gomez, 2019) and confirm something not much studied before, namely, the role that the identification with male dominance and violence against women has in male adolescent sexual harassment online, although it is likely that some of these males are unaware of the destructive nature of the behaviors of harassment that they perform online, as also found in Brown et al. (2020).

In the four risk conditions related to the use of digital devices (PIU and the three factors of risk behaviors online), the *non-violent* group presented lower scores than the two groups corresponding to abusive behaviors, between which there was no significant differences. The multinomial logistic regression analysis results showed that PIU has no predictive significance, and that the three factors of risk behaviors online contribute to the prediction of membership of the group associated with *sexual harassment online*, and especially risky sexual online behaviors. These behaviors also help to predict membership of the group responsible for *abuse in the three contexts*. To understand this result, it should be remembered that this factor is formed of three risky sexting behaviors and three more on interacting with someone you have met online. As Calvete et al. (2021) suggest, adolescents who carry out sexual harassment online may have learned to normalizing these behaviors by online interaction with strangers who perpetrate sexual solicitations as part of their persuasion strategy, justifying and portraying online sexual behaviors as natural. The results of the study carried out in Spain by Ferragut et al. (2021) on child sexual abuse remembered by adults aged 18–74 reflect a high prevalence (24.5%) of exposure to pornographic material by adults during male childhood, higher than that observed among women (12.4%), results that help explain a greater tendency toward normalization of these behaviors among boys, as also found in Calvete et al. (2021). Therefore, programs aimed at adolescents to prevent violence against women need to include activities that foster the rejection of behaviors that lead to sexual harassment online, inculcating the idea that it can seriously damage the women subjected to this type of conduct. The difficulty faced by many girls in rejecting these online interaction behaviors, as detected by Mishna et al. (2021) in research conducted with discussion groups, could also relate to the normalization of the sexual harassment online displayed by boys against girls.

The fact that these results on male adolescent sexual harassment online occur within a macro-system such as Spain, where many important advances in the fight against DVAW have been made (Díaz-Aguado et al., 2021), goes to show that such gains coincide with the emergence of new forms of violence against women by means of digital devices, which also need to be countered by working on the main risk conditions, such as the identification with male dominance and violence against women and risky sexual online behaviors.

As expected, the group corresponding to *abuse in the three contexts* revealed levels of self-esteem that were lower than in the other two groups, and this variable has a significant predictive value for membership of this group. This is in line with results in other studies on adult males (Gallagher and Parrott, 2011), and with adolescents aged 14–20 (Díaz-Aguado et al., 2011, 2013, 2021), yet differs from results for younger adolescents (Foshee et al., 2001; Merino et al., 2021). This shows that, as in the meta-analysis on self-esteem and aggression by Teng et al. (2015) it was found, the relation between these two variables can vary with age and/or level of education of adolescents. This could also explain the different results obtained here and in the study by Merino et al. (2021), both in Spain, on the predictive value of the stress caused by the undermining of the traditional male stereotype arising from situations of subordination to women (MGRS-WS). The study by Merino et al. (2021) was carried out with adolescents aged 13–16 in compulsory secondary school education, in which the girls performed better academically than the boys, who could thus experience frequent situations of subordination to women. This study was carried out with adolescents aged 14–20 across a range of levels of education in which there was little difference in academic performance between boys and girls, according to results in Díaz-Aguado et al. (2021).

Along with the strengths of this study (a study of male adolescent violence against women from the perspective of boys who perpetrate it in three contexts, with a large representative sample and the use of a non-violent group of adolescents as reference group), the research also has some limitations that are important to consider when interpreting the results. First, as the data are based on responses from self-reporting, they should be supplemented by other qualitative procedures. In this sense, future research could use focus groups to overcome the social desirability bias that self-reporting implies, and make it possible to know to what extent the boys who admit to carrying out online sexual harassment behaviors are aware of the damage they can cause to the women who are on the receiving end of them. Second, a longitudinal methodology is needed to investigate the evolution of the problems detected here. Third, it would be convenient to investigate how the different types of male violence studied here with boys who perpetrate them are combined in terms of the perspective of the adolescent girls who suffer them as victims, and what risk and protection conditions (both for women and men) must be considered for its prevention.

## Data Availability Statement

The raw data supporting the conclusions of this article will be made available by the authors, without undue reservation.

## Ethics Statement

The studies involving human participants were reviewed and approved by Committee of Preventive Psychology Unit UCM. Written informed consent to participate in this study was provided by the participants’ legal guardian/next of kin.

## Author Contributions

MD-A have conducted research, including data collection, theory development, literature search, and discussion. RM-A contributed to research design construction and validation of measures, data collection, method, data analysis, and draft preparation. Both authors contributed to the article and approved the submitted version.

## Conflict of Interest

The authors declare that the research was conducted in the absence of any commercial or financial relationships that could be construed as a potential conflict of interest.

## Publisher’s Note

All claims expressed in this article are solely those of the authors and do not necessarily represent those of their affiliated organizations, or those of the publisher, the editors and the reviewers. Any product that may be evaluated in this article, or claim that may be made by its manufacturer, is not guaranteed or endorsed by the publisher.
